# Sarcomatoid and Unclassified Renal Neoplasms: Histology, Immunology, and Genomics

**DOI:** 10.7759/cureus.97268

**Published:** 2025-11-19

**Authors:** Hussein Qasim, Anas Hayajneh, Hamza Abuuqteish, Karis Khattab, Matteo Luigi Giuseppe Leoni, Giustino Varrassi

**Affiliations:** 1 Department of Pathology and Laboratory Medicine, Jordan University of Science and Technology, Irbid, JOR; 2 Department of Clinical Sciences, Jordan University of Science and Technology, Irbid, JOR; 3 Department of Medical and Surgical Sciences and Translational Medicine, Sapienza University, Rome, ITA; 4 Pain Medicine, Fondazione Paolo Procacci, Rome, ITA

**Keywords:** bap1, cdkn2a, dedifferentiation, immunohistochemistry, molecular profiling, pax8, sarcomatoid renal cell carcinoma, tp53, unclassified renal cell carcinoma

## Abstract

Sarcomatoid and unclassified renal cell carcinomas (RCCs) represent diagnostically and clinically challenging entities characterized by aggressive biology and poor prognosis. Sarcomatoid transformation denotes high-grade dedifferentiation that may arise from any RCC subtype and is marked histologically by malignant spindle cells forming fascicles devoid of epithelial architecture. These tumors exhibit pronounced nuclear pleomorphism, necrosis, and frequent mitoses. Immunohistochemistry confirms their epithelial origin through markers such as cytokeratin, epithelial membrane antigen (EMA), and PAX8, while programmed death-ligand 1 (PD-L1) and p53 overexpression reflect underlying molecular alterations and therapeutic relevance. Unclassified RCCs, by contrast, encompass tumors that defy standard classification due to mixed or ambiguous morphology; however, recent molecular advances have redefined many cases as distinct genetic subsets. Genomic profiling reveals recurrent mutations in TP53, BAP1, and CDKN2A in sarcomatoid RCC, and NF2, SETD2, or ALK/NTRK fusions in unclassified RCC, indicating convergent pathways of dedifferentiation, chromatin remodeling, and cell-cycle dysregulation. Clinically, both tumor groups correlate with advanced stage, rapid progression, and resistance to vascular endothelial growth factor (VEGF)-targeted therapies, though immune checkpoint inhibitors show emerging benefit, especially in PD-L1-positive sarcomatoid cases. Integrating histopathologic, immunophenotypic, and molecular features enhances diagnostic accuracy, prognostic stratification, and the identification of actionable targets in these high-risk renal neoplasms.

## Introduction and background

Renal cell carcinoma (RCC) is a heterogeneous group of malignancies arising from the renal tubular epithelium, with multiple distinct subtypes defined by histologic and molecular features [1]. Over recent decades, the classification of RCC has expanded to include entities such as clear-cell RCC, papillary RCC (types 1 and 2), chromophobe RCC, translocation-associated RCC, collecting duct carcinoma, and others [2]. Even so, a subset of tumors does not fit any established category and is designated as unclassified renal cell carcinoma (uRCC( [3]. Additionally, RCCs of any type can undergo high-grade transformation into a spindle-cell malignancy resembling sarcoma, a phenomenon known as sarcomatoid differentiation [4]. Sarcomatoid renal cell carcinoma (sRCC) was historically considered a separate subtype called “renal carcinosarcoma,” but modern understanding recognizes it as a morphologic change that can supervene in any RCC subtype [5]. It is essentially a form of dedifferentiation: well-documented cases show an underlying conventional RCC (often clear-cell carcinoma or others) with areas that have transformed into spindle-shaped, high-grade malignant cells [6]. Sarcomatoid features can be seen in all adult RCC subtypes, most frequently in clear-cell RCC (owing to its overall predominance) but also in papillary, chromophobe, and others [6]. Notably, sarcomatoid change is observed in roughly 5% of all RCC cases, and up to about 10-20% of advanced-stage or metastatic RCCs, underscoring its relevance in clinical practice [5]. The presence of any sarcomatoid component is regarded as a grade 4 (highest grade) tumor in current grading systems, reflecting its strong correlation with aggressive behavior [7]. uRCC is defined by the World Health Organization (WHO) as a diagnostic category of last resort for renal carcinomas that cannot be assigned to a recognized subtype after thorough evaluation [8]. Approximately 2-6% of adult RCCs fall into this unclassified group at experienced centers [9]. These cases often have atypical or mixed histologic features that overlap multiple categories or represent patterns not accounted for in existing classifications [10]. For example, a single tumor might display areas resembling clear-cell RCC combined with papillary architecture, or exhibit unusual features such as mucin production or extensive stromal elements, precluding a single definitive classification [11]. Some unclassified tumors are high-grade malignancies (including those with pure sarcomatoid or rhabdoid morphology and no identifiable epithelial component), whereas others are low-grade neoplasms that simply do not match known benign or malignant renal tumors [12]. In pediatric patients, RCC is rare but shows a different distribution of subtypes; notably, translocation-associated RCC (TFE3 or TFEB rearranged) is more common in children and young adults [13]. Pediatric RCCs also have a higher tendency to be unclassified; historically, up to 20-25% of pediatric RCC cases were unclassifiable on morphology alone, suggesting the presence of distinct entities later recognized (such as MiT family translocation RCC and renal medullary carcinoma) [13]. Addressing both adult and pediatric perspectives, we will explore the histopathologic spectrum of uRCC and how emerging molecular techniques are helping reclassify many of these cases [13]. Given their highly aggressive nature and limited response to standard therapies, early recognition and accurate classification of these variants are critical for optimizing patient management and identifying candidates for emerging immunotherapeutic strategies.

## Review

Methods

This review was conducted as a narrative synthesis following the general principles of the Preferred Reporting Items for Systematic Reviews and Meta-Analyses (PRISMA) 2020 framework for transparency in literature-based research. A comprehensive search of the PubMed, Scopus, and Google Scholar databases was performed to identify relevant English-language publications from January 2000 to October 2025. The search strategy incorporated combinations of the following keywords and Medical Subject Headings (MeSH) terms: “sarcomatoid renal cell carcinoma,” “unclassified renal cell carcinoma,” “renal neoplasms,” “molecular alterations,” “immunohistochemistry,” “checkpoint inhibitors,” and “genomic profiling.”

Reference lists of retrieved articles and recent reviews were manually screened to capture additional relevant studies. Only peer-reviewed original articles, review papers, and clinical or translational studies providing histopathologic, immunophenotypic, molecular, or therapeutic insights into sarcomatoid and unclassified renal neoplasms were included. Case reports and case series were considered when they contributed novel diagnostic or genomic information. Non-English publications, conference abstracts, and studies without verifiable data were excluded.

The selection process involved independent screening by the authors based on titles and abstracts, followed by full-text evaluation for relevance. Data extraction focused on histopathologic features, immunohistochemical markers, molecular alterations, and clinical outcomes, with special attention to studies describing genetic drivers, immune checkpoint expression, and treatment responses.

Given the heterogeneous and descriptive nature of the available evidence, no quantitative synthesis, meta-analysis, or statistical pooling was performed. Instead, findings were integrated qualitatively to provide a structured overview of current knowledge across morphologic, immunologic, and genomic domains. The methodology emphasizes interpretive synthesis rather than numerical aggregation, consistent with the aims of a narrative review.

Histopathology of sarcomatoid and unclassified renal cell carcinoma

Sarcomatoid Differentiation in Renal Cell Carcinoma

Histologically, sRCC is characterized by malignant spindle cells resembling a high-grade sarcoma [5]. These spindle cells typically form intersecting fascicles or sheets lacking the usual architectural patterns of RCC [14]. Cellular pleomorphism is often marked, as the cells may be elongated with atypical, hyperchromatic nuclei; frequent mitoses (including atypical forms) are seen, and regions of coagulative tumor necrosis are common [15]. In some cases, the sarcomatoid component can exhibit diverse appearances, most commonly resembling fibrosarcoma or malignant fibrous histiocytoma (undifferentiated pleomorphic sarcoma) morphology, but it may also show elements of myxoid change or pleomorphic giant cells [16]. Notably, heterologous differentiation (the presence of non-epithelial elements such as malignant bone, cartilage, or muscle) can occur in sRCC [17].

A defining feature of sarcomatoid areas is the absence or minimal presence of identifiable epithelial architecture [18]. By definition, sRCC arises in a carcinoma, so an epithelial component of conventional RCC should be present somewhere in the tumor; however, in the sarcomatoid regions themselves, glandular or tubular structures are usually lost [7]. Occasionally, careful sampling reveals a transition zone where carcinoma cells merge into sarcomatoid morphology [19]. In many tumors, pathologists can find residual foci of the parent RCC subtype, for instance, areas of clear-cell RCC or papillary RCC, adjacent to the sarcomatoid zones, confirming the origin [20]. Clear-cell RCC is the subtype most often associated with sarcomatoid transformation (accounting for roughly 80% of cases of RCC with sarcomatoid change), followed by chromophobe RCC and papillary RCC [20-22]. This frequency largely reflects the prevalence of those subtypes; it does not imply that clear-cell inherently dedifferentiates more readily, although some studies suggest clear-cell RCCs with certain genetic mutations (e.g., BAP1 mutations) are predisposed to high-grade transformations [23].

**Figure 1:**
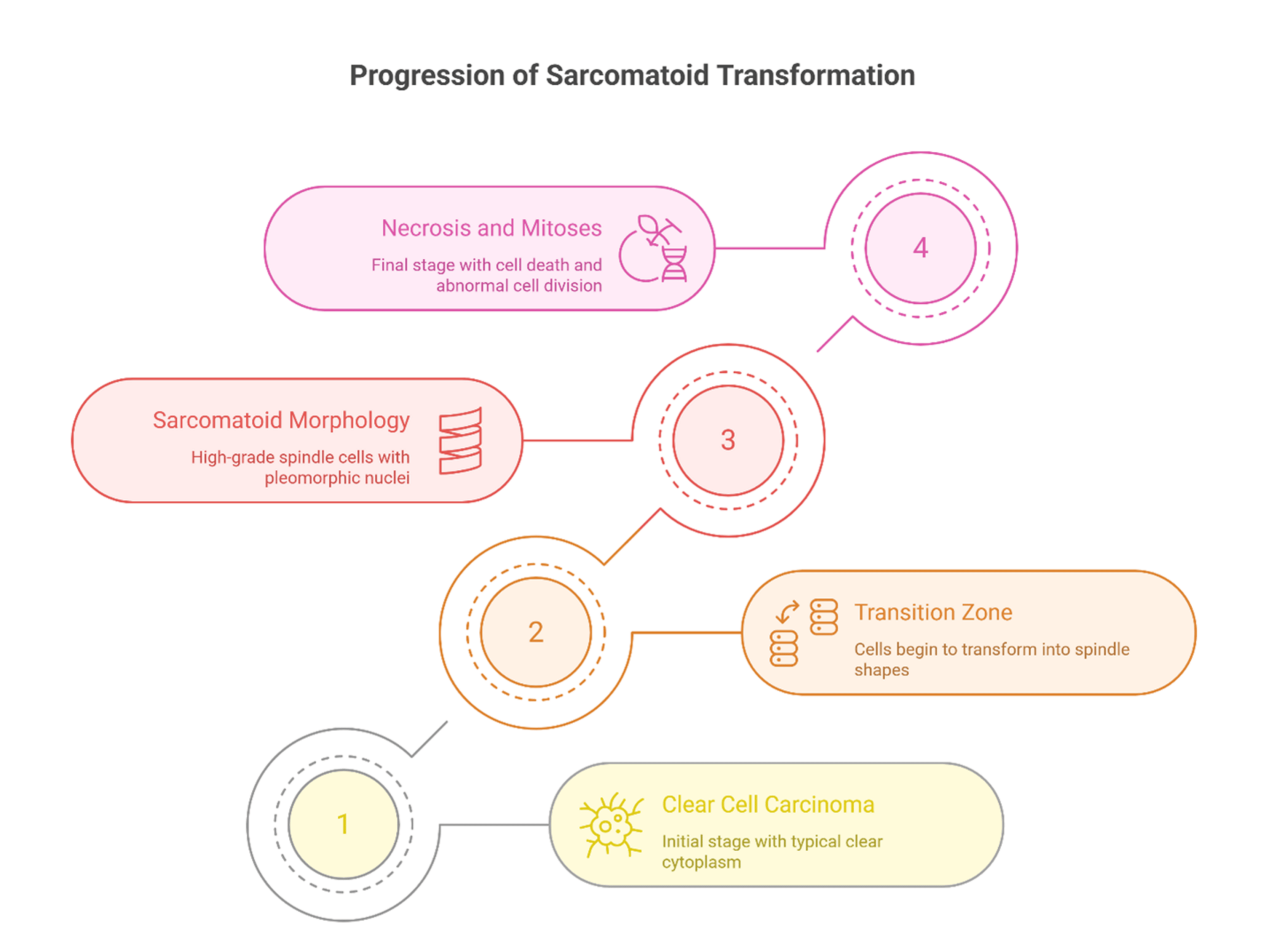
Progression of sarcomatoid transformation Image Credits: Dr. Karis Khattab

Less commonly, sarcomatoid differentiation can occur in more unusual RCC types such as collecting duct carcinoma or even translocation RCC [24]. When a sarcomatoid spindle cell carcinoma is found in the kidney without a clear epithelial component, the differential diagnosis must include a primary renal sarcoma (for example, leiomyosarcoma) or a sarcomatoid urothelial carcinoma of the renal pelvis [25]. Extensive sampling and ancillary studies are often necessary in such cases to establish that it is an RCC with sarcomatoid change rather than a primary sarcoma [26,27]. In practice, if no epithelial elements are detected after thorough examination, these tumors are often categorized as uRCC with sarcomatoid features, acknowledging the uncertainty of their origin (though immunoprofile usually helps, as discussed later) [28]. Occasionally, careful sampling reveals a transition zone where carcinoma cells merge into sarcomatoid morphology (Figure 1).

Virtually all sRCCs are assigned Fuhrman/ISUP (International Society of Urological Pathology) nuclear grade 4 because of the marked nuclear atypia in the spindle cells [28]. Tumor necrosis is present in a high proportion of cases (studies report necrosis in ~80-90% of RCCs with sarcomatoid areas), which further indicates aggressive biology [21,29]. Microvascular invasion can also be seen [30]. Another related form of dedifferentiation in RCC is rhabdoid change, wherein tumor cells become polygonal with eccentric nuclei and hyaline eosinophilic cytoplasmic inclusions (resembling rhabdoid tumor of infancy) [31].

Rhabdoid features, like sarcomatoid ones, are associated with high-grade and often coexist with sarcomatoid areas or occur in the same clinical spectrum of aggressive RCC [32]. If rhabdoid cells are present, they are also considered grade 4 [33]. It is worth noting that rhabdoid morphology retains some epithelial characteristics microscopically (cells can still form cohesive clusters), unlike the overtly spindle-cell sarcomatoid pattern [34].

The histologic spectrum of sarcomatoid transformation is summarized in Table 1.

**Table 1:** Histologic and clinical features of sRCC sRCC, sarcomatoid renal cell carcinoma; RCC, renal cell carcinoma; EMA, epithelial membrane antigen

Feature	Description	Typical findings
Histologic appearance	Biphasic pattern with both epithelial and spindle components [35]	Malignant spindle cells resembling high-grade sarcoma [35]
Common epithelial origin	Clear cell RCC (most frequent), papillary, chromophobe, collecting duct [36]	Variable depending on underlying subtype [36]
Nuclear features	Pleomorphic, hyperchromatic, high mitotic index [37]	Often includes atypical mitoses [37]
Necrosis	Common [37]	Associated with poor prognosis [38]
Immunoprofile	Positive for cytokeratin, vimentin; variably positive for EMA [39]	Loss of typical RCC subtype markers possible [39]

Unclassified Renal Cell Carcinoma

uRCC does not refer to a single morphology but rather encompasses any renal carcinoma that cannot be placed into an existing subtype [40]. The histologic appearance of uRCCs is therefore quite heterogeneous [41]. In some cases, a tumor may exhibit mixed patterns corresponding to more than one known subtype, for example, a mixture of clear-cell areas and papillary structures or combined features of chromophobe and oncocytoma-like cells [42]. When a single predominant pattern is lacking and multiple divergent patterns are present, classification becomes ambiguous, and the tumor may be designated unclassified [43]. In other cases, the tumor’s morphology is highly unusual or unique [43]. Reported examples of uRCC include tumors with mucinous or signet-ring cell features (mucin production is very uncommon in typical RCC subtypes), tumors with prominent stromal components (mimicking biphasic tumors where epithelial elements are intermixed with spindle cell stroma in a way that does not fit carcinosarcoma or mixed epithelial-stromal tumor categories), or tumors composed of cells that do not resemble any normal renal cell or any well-defined tumor type [44]. Some uRCCs have predominantly eosinophilic (oncocytic) cells but lack the characteristic nuclei or architecture of oncocytoma or chromophobe RCC [45]. Others may show spindle cell or sarcomatoid morphology without an obvious epithelial counterpart or display a tubulopapillary architecture that does not match criteria for papillary RCC [46]. Essentially, any renal carcinoma that evades classification after considering all known entities falls under this umbrella [47].

Given this catch-all nature, uRCC is a diagnosis of exclusion [48]. For example, high-grade unclassified carcinomas could be confused with metastases from other organs (such as anaplastic carcinoma of the lung or elsewhere), so clinical correlation and immunohistochemical confirmation of renal origin are important [49]. The WHO definition of uRCC (2016 and updated 2022 classifications) includes tumors with mixed histologic features (admixed patterns of recognized subtypes), unclassified oncocytic tumors (that do not meet criteria for chromophobe RCC or oncocytoma), pure sRCC without identifiable epithelial elements, and other uncommon patterns such as those with mucin secretion or dual epithelial-stromal differentiation [3,47]. Some specific hereditary RCC syndromes or emerging tumor types can present with unusual morphology and might have been labeled unclassified in the past before their distinct identity was known, for instance, RCC associated with fumarate hydratase (FH) gene mutations (formerly an unclassified high-grade tumor, now recognized as FH-deficient RCC) or anaplastic lymphoma kinase (ALK)-rearranged RCC (a rare entity often with morphologies that puzzled pathologists initially) [50]. In children and adolescents, a significant fraction of RCCs were historically unclassified, but many of these have since been identified as translocation carcinomas involving TFE3/TFEB or as renal medullary carcinoma (a rare tumor almost exclusive to patients with sickle cell trait) [51]. Thus, the unclassified category is continuously shrinking as new entities are defined, but it remains an important category for cases with truly ambiguous features [52].

Many uRCCs reported from tertiary centers skew toward high-grade tumors, large, aggressive neoplasms that likely correspond to as-yet-undefined molecular categories [53]. High nuclear grade, sarcomatoid and/or rhabdoid features, and tumor necrosis are frequently observed in uRCC cases, correlating with their generally poor outcomes [54]. However, there are also low-grade uRCCs that may behave more indolently [55]. An example of a low-grade unclassified scenario is a predominantly oncocytic tumor in a young patient that does not fit chromophobe RCC or oncocytoma criteria; some of these have later been shown to have unique genetic alterations (for instance, tumors in young patients with eosinophilic morphology and unclassified histology have been found in some studies to carry TSC/mTOR pathway mutations, suggesting a link to the eosinophilic solid and cystic RCC recently described) [56].

**Figure 2:**
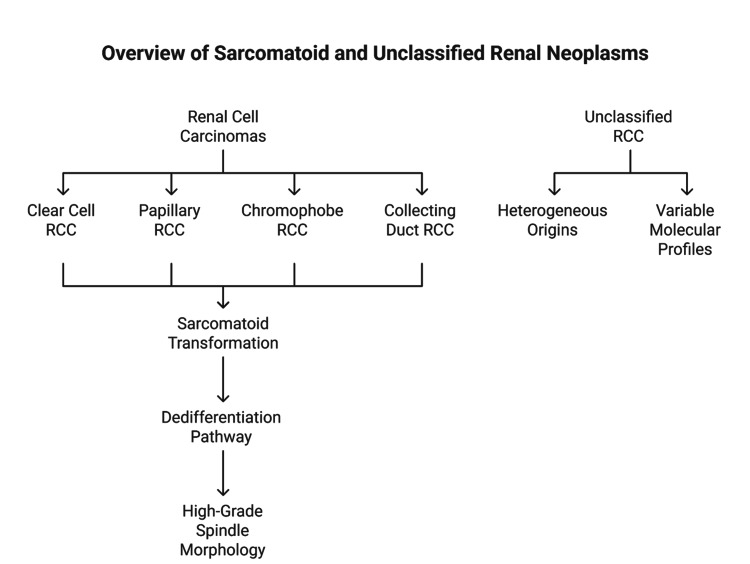
Overview of sarcomatoid and unclassified renal neoplasms RCC, renal cell carcinoma Image Credits: Dr. Karis Khattab

Sarcomatoid transformation represents a dedifferentiation process arising from various RCC subtypes, whereas uRCCs comprise morphologically heterogeneous and molecularly variable neoplasms (Figure 2).

Immunohistochemistry

Immunohistochemical analysis plays a pivotal role in the evaluation of both sarcomatoid and uRCCs [57]. It confirms the renal epithelial origin of an undifferentiated tumor, helps distinguish sarcomatoid carcinoma from primary renal sarcoma, and provides valuable clues for subclassification when morphology is inconclusive [58].

In sRCC, the spindle-cell component can closely mimic a true sarcoma on light microscopy [59]. Sarcomatoid tumor cells usually express broad-spectrum cytokeratins (pancytokeratin) and epithelial membrane antigen (EMA), at least focally [37]. PAX8, a nuclear transcription factor specific for renal and Müllerian epithelium, is especially useful, as its expression in spindle-cell lesions strongly supports renal carcinoma origin [60]. The combined demonstration of PAX8 and cytokeratin positivity essentially confirms sarcomatoid carcinoma rather than a primary sarcoma [61]. Vimentin expression is nearly universal but nonspecific, since it is shared by both sarcomas and epithelial RCCs [62]. Other traditional RCC markers, such as CD10 and carbonic anhydrase IX (CAIX), may persist in sarcomatoid areas but often in a reduced or patchy fashion compared with the parent carcinoma [63]. For instance, clear-cell RCC typically shows diffuse CAIX positivity, whereas its sarcomatoid areas exhibit only focal staining [64]. Similarly, sarcomatoid transformation of chromophobe RCC may result in loss of CK7 and c-KIT (CD117) expression [65]. Overall, the sarcomatoid component tends to retain only the core epithelial markers while losing lineage-specific differentiation antigens [66].

sRCC also commonly shows overexpression of p53 protein, correlating with underlying TP53 mutation, and strong PD-L1 (programmed death-ligand 1) expression on tumor and immune cells [67]. The latter finding is clinically relevant, as PD-L1 positivity has been associated with responsiveness to immune checkpoint inhibitors [68]. A high Ki-67 proliferation index is typical, reflecting the brisk mitotic activity of these tumors [69].

In uRCC, immunohistochemistry is equally essential, though by definition these tumors lack a characteristic profile [70]. Instead, a broad panel is used to confirm renal origin and systematically exclude recognized RCC subtypes [70]. Confirmation of renal epithelial differentiation is achieved with markers such as PAX8, PAX2, and pancytokeratins [27]. Most uRCCs are at least focally positive for these markers; if PAX8 is absent, metastatic carcinoma or sarcoma should be reconsidered [71]. Subtype-associated markers are then applied based on morphology: CAIX and CD10 for clear-cell features, CK7 and AMACR (α-methylacyl-CoA racemase) for papillary morphology, and CK7 with c-KIT (CD117) for chromophobe-like or eosinophilic tumors [72].

Because translocation RCCs often masquerade as unclassified tumors, especially in younger patients, immunostains for TFE3 and TFEB are routinely performed; nuclear overexpression suggests MiT (microphthalmia-associated transcription) family translocation RCC, which can be confirmed molecularly [73]. These tumors may also express melanocytic markers such as Melan-A or cathepsin K [74]. In high-grade uRCCs, particularly in young patients or those with sickle cell trait, INI1 (SMARCB1) staining is critical to identify renal medullary carcinoma or related rhabdoid tumors [75]. For oncocytic tumors that do not fit the chromophobe-oncocytoma spectrum, SDHB immunostaining can detect succinate dehydrogenase (SDH) deficiency, defining SDH-deficient RCC [76]. Similarly, high-grade papillary or eosinophilic tumors with bizarre nuclei should be evaluated for FH loss and 2-succinyl-cysteine (2SC) accumulation to diagnose FH-deficient RCC, a hereditary leiomyomatosis-associated variant [77].

Only after comprehensive immunohistochemical and molecular assessment should a tumor be designated as unclassified [78]. Many uRCCs display mixed or discordant immunoprofiles, for example, coexpression of CAIX and CK7 or patchy reactivity for CD10 and CK7, without fitting any established subtype [79,80]. In such cases, the diagnosis rests on exclusion [78].

A panel of immunohistochemical markers and their diagnostic implications is shown in Table 2.

**Table 2:** Immunohistochemical markers in sarcomatoid and unclassified renal neoplasms PAX8, paired box gene 8; S100, soluble protein 100; H3K27me3, trimethylation of histone H3 at lysine 27; AE1/AE3, cytokeratin antibody cocktail AE1/AE3; Vimentin, intermediate filament protein; Desmin, muscle-specific intermediate filament protein

Marker	Expression pattern	Diagnostic utility	Notes
Vimentin [81]	Positive in both epithelial and sarcomatoid areas	Supports mesenchymal differentiation	Non-specific but consistent
Cytokeratin (AE1/AE3) [5]	Often retained in sarcomatoid component	Confirms epithelial origin	Loss may occur in high-grade areas
PAX8 [61]	Usually positive	Indicates renal epithelial lineage	May be weak in sarcomatoid foci
S100, Desmin [82]	Focally positive	Helps in differential diagnosis	Non-specific expression
H3K27me3 [83]	Loss in some unclassified and sarcomatoid tumors	Marker of aggressive epigenetic dysregulation	Emerging prognostic tool

Genomic and molecular features

Molecular studies have provided key insights into the genetic alterations driving sarcomatoid transformation and uRCC, describing their aggressive biology and, in some cases, suggesting therapeutic targets [84]. In sRCC, dedifferentiation is thought to occur through stepwise tumor progression in which an existing RCC acquires additional driver mutations [23]. Among the most consistently implicated genes is TP53, encoding the p53 tumor suppressor [23]. TP53 mutations are markedly enriched in sarcomatoid components, often absent from the carcinoma areas, indicating a late event driving dedifferentiation [85]. Loss of p53 function promotes genomic instability and accelerates further mutation accumulation, consistent with the highly pleomorphic phenotype [86]. Consequently, sRCCs display complex karyotypes with multiple chromosomal gains and losses, far more aberrant than conventional RCCs [87]. Another frequent alteration involves CDKN2A (9p21), encoding p16 INK4a [88], with homozygous deletions or inactivating events disrupting the retinoblastoma (RB) pathway and promoting uncontrolled proliferation [88]. Co-deletion of the adjacent CDKN2B (p15) is common, and these alterations, rare in early-stage RCC, are enriched in aggressive tumors [89]. In clear-cell RCC, from which most sRCCs arise, early driver events include 3p loss and VHL mutation, activating hypoxia and vascular endothelial growth factor (VEGF) pathways [90].

Sarcomatoid clear-cell RCC typically retains these background mutations (VHL, PBRM1, SETD2) but acquires additional lesions such as TP53 and BAP1 mutations [91]. BAP1, a 3p tumor suppressor, correlates with high-grade and sarcomatoid or rhabdoid morphology, while PBRM1 and BAP1 mutations are largely mutually exclusive, BAP1-mutant tumors tending toward greater aggressiveness [92]. Additional changes include NF2 loss, affecting the Hippo pathway, and activation of PI3K/AKT/mTOR signaling via MTOR or PTEN mutations [93]. Large-scale profiling efforts, including The Cancer Genome Atlas (TCGA), confirm that RCCs with sarcomatoid or rhabdoid features form a distinct molecular cluster characterized by chromatin remodeling gene loss (BAP1, ARID1A), cell-cycle dysregulation (TP53, CDKN2A), and an inflammatory immune gene signature with high PD-L1 expression and macrophage/T-cell infiltration [94]. Conversely, these tumors show reduced angiogenic signaling, correlating with their poor response to VEGF-targeted therapies [95]. Exome sequencing of matched tumor regions confirms clonality between carcinoma and sarcomatoid components, with the latter harboring additional unique mutations, supporting a model of branched evolution and late-stage dedifferentiation akin to anaplastic transformation in other organs [96].

In uRCC, molecular analysis is beginning to unravel the heterogeneity of this once-ambiguous category [96]. Recent sequencing studies have revealed recurrent NF2 mutations in 15-20% of cases, often associated with high-grade morphology and Hippo pathway inactivation [97]. Mutations in chromatin-modifier genes such as SETD2 and KMT2C (MLL3) are also common, paralleling pathways known from clear-cell RCC but in novel combinations [98]. MTOR, TSC1, and TSC2 alterations occur in subsets with eosinophilic histology, potentially overlapping with entities like eosinophilic solid and cystic RCC [99]. BAP1 mutations appear in some high-grade unclassified cases lacking clear-cell features, suggesting shared pathogenic mechanisms with aggressive clear-cell RCC [100]. Notably, many uRCCs lack VHL mutations and chromosome 3p loss, distinguishing them from atypical clear-cell RCC and indicating separate molecular lineages [101]. Genomic profiling has redefined numerous “unclassified” cases as specific molecular entities [102]. Tumors harboring ALK or NTRK fusions can now be diagnosed as ALK- or NTRK-rearranged RCC and treated with targeted inhibitors such as crizotinib or larotrectinib [103]. Similarly, RNA sequencing may uncover TFE3 or TFEB fusions, identifying translocation RCC that evaded detection by immunohistochemistry [104]. Comprehensive sequencing panels can reclassify up to 70-75% of uRCCs by revealing actionable or defining mutations, including MET, NF2, FH, and SDH alterations [102].

Clinical implications and prognosis

The diagnoses of sRCC and uRCC carry major clinical significance because both entities are strongly associated with aggressive biological behavior and adverse outcomes [4,105]. Compared with conventional RCC subtypes, they portend poorer survival and require distinct therapeutic considerations and vigilant follow-up strategies [106].

Sarcomatoid differentiation is a well-established marker of high-grade, aggressive disease and may occur in any RCC subtype [106]. Patients with sRCC often present at an advanced stage, typically with large, locally invasive tumors (T3-4) that frequently involve perinephric tissues, the renal vein, or adjacent organs [107]. Regional lymph node metastases and distant spread are common at diagnosis, with metastatic patterns resembling those of conventional RCC but characterized by more rapid progression and heavier disease burden [108]. Numerous studies have confirmed sarcomatoid change as an independent adverse prognostic factor, even after controlling for tumor stage and grade [38]. Patients with localized sRCC have a significantly higher risk of recurrence after nephrectomy than those with stage-matched, non-sarcomatoid tumors [109]. Historically, before the advent of immune checkpoint blockade, the median survival for metastatic sRCC was approximately six to 12 months, compared with two to three years for metastatic clear-cell RCC without sarcomatoid features [110].

The extent of sarcomatoid transformation correlates with outcome, with tumors containing over 50% spindle cell components showing particularly poor survival [111]. Nevertheless, even a minimal sarcomatoid component (≥5%) carries prognostic importance [111]. Additional morphologic features such as tumor necrosis, rhabdoid differentiation, and vascular invasion further compound the unfavorable prognosis [111]. Clinically, recognition of sarcomatoid differentiation is essential because it influences therapeutic decision-making [111]. These tumors exhibit relative resistance to tyrosine kinase inhibitors (TKIs) and other VEGF-targeted therapies that revolutionized RCC management in the 2000s [112]. Historically, patients with sRCC were excluded from pivotal trials and showed inferior outcomes when treated with VEGF-directed agents such as sunitinib or pazopanib, reflecting the distinct biology of these tumors [110]. Although modern immunotherapy has improved outcomes in a subset of patients, sRCC remains a high-risk variant requiring aggressive multimodal management and close surveillance [113].

uRCC, by contrast, represents a heterogeneous group of tumors that fail to fit existing diagnostic categories [3]. Prognosis varies widely, but most uRCCs behave aggressively and are associated with poor clinical outcomes [78]. Population-based analyses and institutional series consistently show that uRCC carries one of the lowest overall survival rates among all RCC subtypes, even after adjustment for stage [114].

Several clinicopathologic factors influence prognosis in uRCC, including tumor size, presence of necrosis, and mitotic activity [115]. Even localized unclassified tumors show a higher risk of postoperative recurrence, underscoring the need for close follow-up [116]. Pediatric uRCCs are exceedingly rare, but molecular analyses frequently reclassify these tumors as translocation RCC or medullary carcinoma [117]. Translocation RCC generally has an intermediate prognosis, whereas renal medullary carcinoma is among the most aggressive renal malignancies, often fatal within one year [118]. For this reason, pediatric unclassified cases should be evaluated at specialized centers and included in collaborative registries to refine classification and outcome prediction [119].

Both sarcomatoid and uRCCs have historically been refractory to standard therapy, though recent developments have begun to alter this outlook [120]. sRCC shows poor responsiveness to VEGF-targeted TKIs, and attempts to treat it with cytotoxic chemotherapy regimens such as gemcitabine plus doxorubicin yielded limited benefit [121]. The major therapeutic advance has been the advent of immune checkpoint inhibitors [121]. Combination regimens involving PD-1 and CTLA-4 blockade (e.g., nivolumab plus ipilimumab) have produced markedly superior response rates compared with sunitinib in patients with sarcomatoid differentiation, including occasional complete remissions, an outcome previously unprecedented in this setting [122]. Recent combination immunotherapy trials, such as CheckMate 9ER and COSMIC-313, have shown improved response rates and survival in patients with sarcomatoid differentiation, emphasizing the role of dual checkpoint blockade in these aggressive RCC variants [123].

For uRCC, evidence remains limited because of the absence of dedicated clinical trials, and management is often empirical [124]. Treatment is typically modeled after the most closely resembling RCC subtype: VEGF or mesenchymal-epithelial transition (MET) inhibitors may be considered for papillary-like morphology, while TKI- or immunotherapy-based regimens are used for clear-cell-like or high-grade tumors [124]. Trials such as ASPEN and ESPN have shown modest efficacy of sunitinib in non-clear-cell RCC, though outcomes remain inferior to clear-cell counterparts [125]. Immunotherapy has shown encouraging activity in non-clear-cell cohorts, including unclassified cases, particularly those with high PD-L1 expression or sarcomatoid components [126]. Ongoing studies are specifically evaluating dual checkpoint blockade in rare RCC subtypes [127]. Furthermore, molecular profiling can occasionally identify actionable genomic alterations, such as ALK or NTRK fusions, that allow the use of targeted agents like crizotinib or larotrectinib, offering the potential for remarkable responses in otherwise refractory disease [128].

Emerging strategies

The therapeutic and research landscape for aggressive RCCs, particularly sarcomatoid and unclassified types, is rapidly evolving toward precision medicine and immune-based strategies [113]. Advances in immunotherapy have significantly altered the outlook for sRCC, with ongoing trials evaluating combinations of PD-1 inhibitors and VEGF-targeted TKIs (e.g., pembrolizumab plus axitinib, nivolumab plus cabozantinib) that may enhance immune responsiveness by modulating the tumor microenvironment [129]. Novel approaches such as adoptive cell therapy, alternative checkpoint blockade (LAG-3, TIGIT), and cytokine-based treatments are also under investigation for immunotherapy-resistant cases, building on the high PD-L1 expression typical of sarcomatoid tumors [130].

For uRCC, molecular profiling is redefining therapeutic possibilities by identifying actionable targets [131]. Tumors with MET alterations may respond to MET inhibitors, TSC1/TSC2 or MTOR mutations to mTOR inhibitors, and rare ALK or NTRK fusions to their respective targeted agents [132]. Translocation RCCs with TFE3 or TFEB fusions and tumors showing high mutational burden may also benefit from tailored or immunotherapeutic regimens [133]. As genomic testing becomes routine in atypical RCCs, such precision approaches are increasingly feasible, offering diagnostic clarity and therapeutic guidance [134]. Emerging research on epigenetic alterations (BAP1, PBRM1, SETD2, CDKN2A) and tumor-stroma interactions is identifying new drug targets, including DNA damage response and CDK4/6 inhibitors, as well as microenvironment-directed therapies such as CSF1R blockade [135]. Concurrently, advanced molecular diagnostics, mRNA expression profiling, and AI-based histologic classification are improving diagnostic accuracy and helping delineate new molecular subtypes previously deemed “unclassified” [136].

Challenges and future directions

Despite meaningful advances in renal cancer research, sarcomatoid and uRCCs continue to present major diagnostic, biological, and therapeutic challenges [137]. The “unclassified” designation reflects current limitations in tumor categorization, as many lesions exhibit overlapping or borderline features that defy existing criteria [138]. Refinement of molecular diagnostics and the continual evolution of the WHO classification are expected to reduce this category by identifying new molecularly defined subtypes [139]. In sRCC, accurate diagnosis requires thorough sampling, since limited biopsies may capture only the spindle component, risking misinterpretation as primary sarcoma [25]. Standardized pathology reporting and quantification of sarcomatoid components are needed to improve prognostic assessment [140].

At the biological level, the mechanisms driving sarcomatoid or rhabdoid transformation remain incompletely understood [141]. Although recurrent mutations such as TP53 and CDKN2A have been implicated, additional epigenetic and microenvironmental factors likely contribute [142]. Progress will depend on developing robust experimental models and integrating multi-omics data from international collaborations [143]. AI and digital pathology may also help reveal diagnostic patterns in morphologically ambiguous cases [144].

Therapeutically, both sarcomatoid and uRCCs are characterized by aggressive behavior and limited responsiveness to standard regimens [121]. While immune checkpoint inhibitors have improved outcomes for some patients with sRCC, resistance remains common [145]. Broader clinical trial inclusion, molecularly guided therapy, and novel drug combinations are essential to advance care [146]. Emerging biomarkers and liquid biopsy technologies may eventually predict dedifferentiation or treatment response [147].

Future progress will rely on the integration of histologic, immunophenotypic, and molecular insights to achieve more precise classification and individualized treatment. Through collaborative research, innovative trial design, and molecularly informed therapy, the boundaries of “unclassified” RCC will continue to narrow, and sarcomatoid transformation, once a marker of hopeless prognosis, may become a targetable and potentially manageable phenotype.

Emerging molecular and immunologic targets with therapeutic relevance are summarized in Table 3.

**Table 3:** Emerging biomarkers and therapeutic implications PD-L1, programmed death-ligand 1; ICI, immune checkpoint inhibitor; mTOR, mechanistic target of rapamycin; BAP1, BRCA1-associated protein 1; SETD2, SET domain containing 2; HIF-1α, hypoxia-inducible factor 1-alpha; VEGF, vascular endothelial growth factor; uRCC, unclassified renal cell carcinoma

Biomarker	Biological tole	Potential therapy	Clinical relevance
PD-L1 expression [148]	Immune evasion	Immune checkpoint inhibitors	Predicts ICI responsiveness
MTOR mutations [149]	Cell growth and metabolism	mTOR inhibitors	Found in some uRCC
BAP1 and SETD2 loss [150]	Chromatin regulation	Epigenetic therapies (under investigation)	Associated with high-grade transformation
HIF-1α and VEGF [151]	Hypoxia and angiogenesis	Anti-VEGF agents	Common in clear cell–derived cases

## Conclusions

Sarcomatoid and unclassified renal cell neoplasms represent the most aggressive and diagnostically challenging ends of the renal tumor spectrum. Sarcomatoid differentiation reflects high-grade spindle cell transformation that can arise in any RCC subtype, marking a “final common pathway” of tumor dedifferentiation. In contrast, uRCC encompasses morphologically and genetically diverse tumors that defy existing classification criteria. Histologically, sRCC exhibits biphasic morphology with sarcoma-like spindle cells and residual epithelial areas, while uRCC is defined by exclusion of known subtypes. Immunohistochemistry confirms epithelial lineage in sarcomatoid tumors and aids in characterizing unclassified cases, though aberrant marker expression often complicates interpretation. Genomic studies reveal recurrent mutations, including TP53 and CDKN2A losses, underlying the aggressiveness of sRCC, while many unclassified tumors are now being redefined through molecular subtyping. Clinically, both entities portend a poor prognosis and demand multidisciplinary management. The emergence of immune checkpoint inhibitors has improved outcomes in some sRCCs, whereas molecularly guided therapies offer growing promise for uRCC. Ongoing integration of histologic, immunophenotypic, and genomic data will refine classification systems and reduce diagnostic ambiguity. Continued research, clinical trial inclusion, and therapeutic innovation are essential to improving the outlook for patients with these high-grade renal neoplasms.
